# Enemy’s arsenal backfires: pathogen proteases detonate broad-spectrum plant immunity via engineered NLRs

**DOI:** 10.1007/s44154-026-00296-3

**Published:** 2026-03-05

**Authors:** Shan Liu, Yanping Jing, Jian Chen

**Affiliations:** International Genome Center, Jiangsu University, Zhenjiang, 212013 China

Developing crops with broad-spectrum and durable disease resistance is paramount for global food security amidst escalating pathogen evolution and climate change. Plants primarily rely on nucleotide-binding and leucine-rich-repeat immune receptors (NLRs), comprising three subclasses, namely Toll/interleukin-1 receptor domain-containing NLRs (TNLs), coiled-coil (CC) domain-containing NLRs (CNLs), and RESISTANCE TO POWDERY MILDEW 8-like CC (CC_R_) domain-containing NLRs (RNLs), to recognize pathogen effectors and activate defense responses (Liu et al. 2023). However, traditional NLRs normally have narrow-spectrum resistance since a single NLR typically detects only a limited number of effectors from specific pathogens (Cesari 2018). Additionally, pathogens can rapidly evolve to evade this recognition (Valent 2025). As a result, NLR receptors have been a prime target in the program of plant resistance bioengineering and many methodologies, such as domain swapping, decoy engineering, integrated domain engineering, structure-guided, random or targeted mutagenesis have been developed aiming to expand recognition specificity of NLRs (Anbu et al. 2023). However, NLRs modified by methods such as mutagenesis or domain shuffling-modified may lead to deleterious autoimmune phenotypes, and can be easily evaded by rapid pathogen evolution in the field (Białas et al. 2021; De la Concepcion et al. 2021). In addition, methods such as “decoy engineering” require modifying a decoy protein to activate the corresponding NLR, which is best exemplified by PBS1/RPS5, where the protease cleavage site of decoy protein PBS1 is engineered to be able to detected by various protease effectors from diverse pathogens, activating RPS5-mediated disease resistance. However, this approach is constrained in many crops lack RPS5 or analogous NLRs (Kim et al. 2016). Therefore, novel strategies confer crops broader-spectrum and durable resistance is in desire demand.

Recently, a fascinating study from Wang et al. (2025) proposed a novel NLR remodelling approach to create a “smart” immune receptor, which involves a chimeric protein comprising a flexible polypeptide linker, a pathogen-derived protease cleavage site (PCS), and an autoactive NLR (aNLR). The aNLRs are modified proteins by introducing amino acid substitutions in specific sites of normal NLRs, mimicking pathogen effector-mediated NLR activation (Jacob et al. 2021). The N-terminal fused flexible polypeptide (containing tags like HA) suppresses the activity of the aNLR, keeping it in an “off” state. Upon pathogen invasion, its secreted specific protease cleaves the PCS, releasing the free aNLR, which triggers broad-spectrum and durable plant resistance (Fig. 1).

**Fig. 1:**
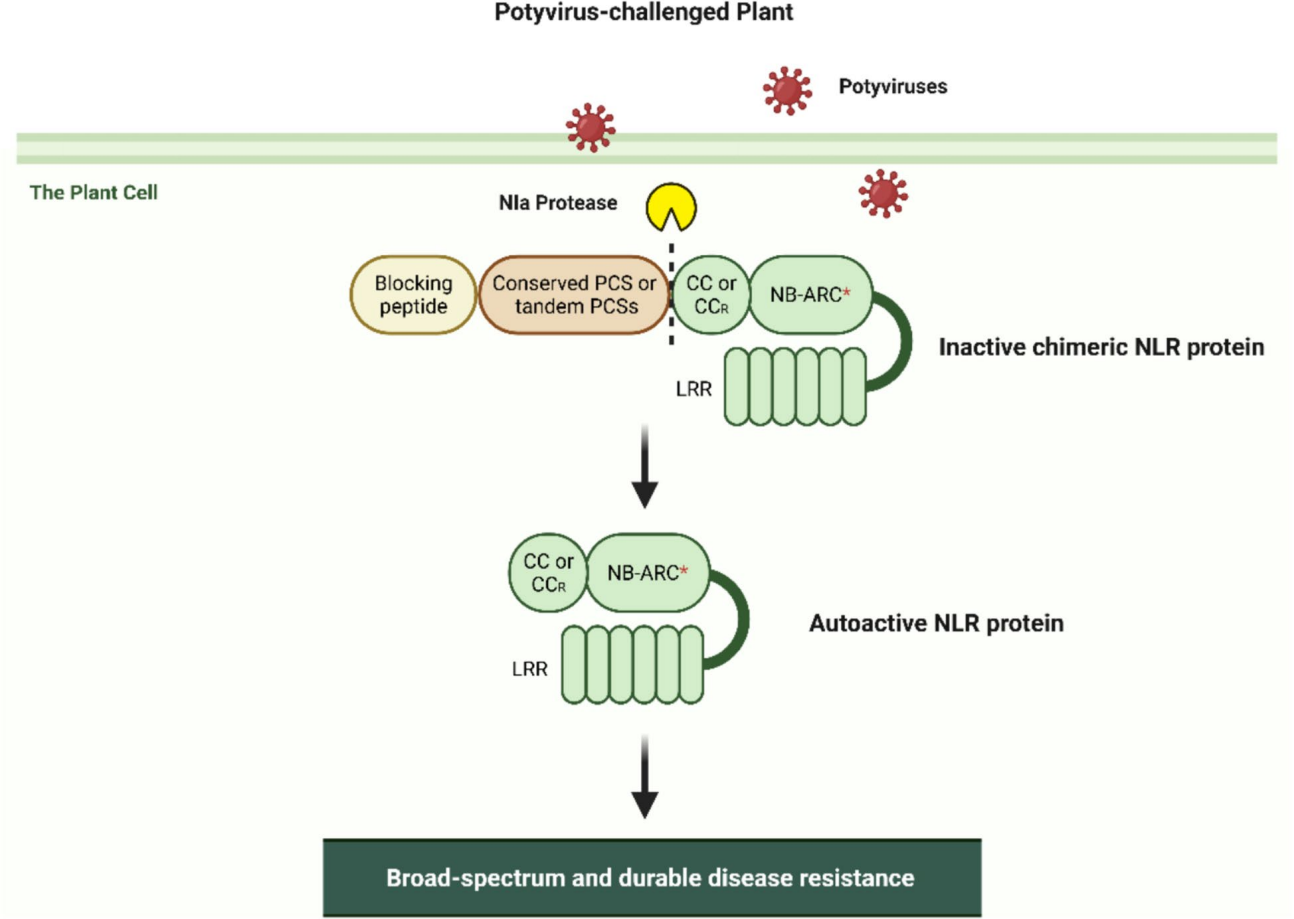
The working model of engineered chimeric NLR activation by potyviral NIa proteases. The engineered chimeric NLR comprises a blocking peptide, a conserved protease cleavage site (PCS) or tandem PCSs from diverse potyviruses, and an autoactive NLR (aNLR). Upon infection, potyvirus-secreted proteases cleave the PCS, releasing the aNLR receptor, which elicits a broad-spectrum immune response against multiple potyviruses.Within the chimeric NLR, CC and CC_R_ are abbreviations of coiled-coil domain and RESISTANCE TO POWDERY MILDEW 8-like CC domain; NB-ARC is a abbreviation of nucleotide-binding domain and the red asterisk represents a substitution conferring autoactivation to NLR; LRR represents leucine-rich repeat-containing domain

This study validated the system using potyviruses as a model. This family includes devastating crop viruses with highly conserved NIa proteases. They fused the conserved PVY(Potato Virus Y) PCS (YEVHHQ↓A) to the N terminus of the autoactive CNL Tm-2^2^, creating chimeric protein HA-PCS^PVY^-aTm-2^2^. These redesigned CNL proteins induced cell death when co-expressed with PVY NIa in *Nicotiana benthamiana.* Immunoblotting assays using anti-HA antibody confirmed the cleavage of both HA-PCS^PVY^-aTm-2^2^ and HA-PCS^PVY^-aTm-2^2^-HA by PVY NIa *in planta.* These results indicate PVY NIa can cleave HA–PCS^PVY^–aTm-2^2^, releasing free autoactive aTm-2^2^ that is able to trigger plant cell death. Furthermore, transgenic *Nicotiana benthamiana* plants expressing this chimeric receptor exhibited significant cell death phenotype upon transient expression of NIa^PVY^ and complete resistance against multiple potyviruses, including PVY, Turnip mosaic virus (TuMV), Pepper mottle virus (PepMoV), chilli veinal mottle virus (ChiVMV) and Plum pox virus (PPV). Replacing the aNLR with the autoactive RNL AtNRG1.1 (HA-PCS^PVY^-aAtNRG1.1) even induced extreme resistance represented by the absence of visible HR lesion and PVY RNA in systemic leaves in transgenic T1 plants when infected by virus strains such as PPV and PepMoV.

Intriguingly, when challenged with Tobacco etch virus (TEV), which has a distinct NIa PCS (ENLYFQ↓G) from XXVXXQ↓A(G/S) consensus motif and PCS^PVY^(YEVHHQ↓A) used in the engineered HA-PCS^PVY^-aNLR (aTm-2^2^ or aAtNRG1.1) proteins, the redesigned receptors failed to confer resistance against infection of this virus. To broaden the resistance specificity, the authors generated an innovative aNLR, HA-PCS^TEV^-PCS^PVY^-aAtNRG1.1, with tandem PCSs containing both TEV and PVY cleavage sites. This receptor was also confirmed to be cleaved by both TEV and PVY proteases, thus triggering cell death. Transgenic plants showed either extreme resistance or complete resistance against PVY, TEV, and four other tested potyviruses used previously. This tandem PCS design overcomes the limitation of NLR recognition specificity on a specific pathogen’s protease, significantly expanding the resistance spectrum against evolutionarily divergent viruses.

To know whether this NLR modified approach applies in crop plants, the authors tested in soybean-soybean mosaic virus (SMV) pathosystem. They constructed HA-PCS^SMV^-aAtNRG1.1 containing the SMV NIa cleavage site PCS^SMV^ (ESVSLQ↓S). Similarly, co-expression assays and the following immunoblots show HA-PCS^SMV^-aAtNRG1.1 was cleaved by NIa^SMV^ in plants. The T₁ transgenic plants inoculated with SMV-eGFP showed no infection symptoms or detectable viral RNA, while exhibiting normal growth and agronomic traits, confirming no growth penalty, suggestive of great potential of applying this method in enhancing disease resistance of important crops.

This study, for the first time, proposes a novel NLR engineering strategy that delivers four transformative advantages over current approaches. Firstly, this approach is straightforward by directly modifying a single NLR receptor gene, contrasting with decoy engineering that necessitates concurrent engineering of both a decoy protein and its cognate NLR (Kim et al. 2016); Secondly, this method can be applied in diverse receptor types including CNL and RNL over multiple plant species, where a single engineered receptor confers resistance against phylogenetically related pathogens. For instance, in this study, a receptor incorporating the conserved PCS^PVY^ confer theoretical protection against around 110 potyviruses; Thirdly, this approach offers plants enhanced resistance durability as resistance breakdown often requires loss-of-function mutations in the pathogen’s protease, which is lethal to pathogens; Fourthly, transgenic plants expressing modified NLRs show high level of resistance against multiple viruses while no obvious growth penalty was observed under greenhouse conditions given that growth-defense trade-off commonly exist in disease resistant plants. Furthermore, this approach will enable application expansion to cross-kingdom pathogens secreting proteases, including bacteria, oomycetes, fungi, and nematodes beyond viruses. More crucially, CRISPR-Cas genome editing can be integrated to modify endogenous NLR genes, circumventing the need for transgenic procedures.

Nevertheless, this technology is accompanied by a number of limitations and questions to be answered. (1) Whether this approach can be applied to generate resistant crops under natural agricultural fields beyond greenhouse conditions without significant growth penalty? (2) Whether autoactivation occur in the absence of the target pathogens? It is possible that abiotic stresses (e.g., drought, heat, or physical injury) or infection by non-target pathogens may induce the expression of certain plant proteases that are not typically active. If these induced plant proteases share sequence similarity with the PCS, they might incidentally cleave the engineered NLR. This could lead to constitutive immune activation even without infection by the intended pathogens. Persistent unintended autoimmunity may consequently result in growth suppression and yield penalties. (3) The authors suggest that tandem PCSs can confer broad-spectrum resistance in plants. However, an important consideration is whether excessive tandems might induce steric hindrance or misfolding of the NLR protein, thereby compromising its resistance function. In addition, would the presence of over 8 amino acids at the N-terminal of NLR affect NLR-meidated resistance? As such, these issues warrant further in-depth investigation in the future studies and applications.

## Conclusion

This research represents a paradigm shift in plant immune engineering from “recognizing specific enemies” to “exploiting a common weapon of the enemy to trigger defense.” The moment the protease carried by the virus cleaves the blocking site of NLR, it unknowingly open its own tomb. By converting pathogen-essential proteases into “suicide switches” that activate plant immunity, this strategy offers a valuable solution for broad-spectrum, durable disease resistance in plants. As field trials progress and more pathogen targets are validated, this “molecular decoy” based smart immune design holds immense promise as a powerful technology for next-generation disease-resistant crops, fortifying the foundation of sustainable agriculture.

## Data Availability

Not applicable.

## Abbreviations

aNLR: Autoactive NLR
ChiVMV: Chilli Veinal Mottle Virus
CNLs: Coiled-coil domain-containing NLRs
ER: Extreme Resistance
HR: Hypersensitive Response
NIa: The nuclear inclusion a
NLRs: Nucleotide-binding and Leucine-rich-repeat immune Receptors
PBS1: AVRPPHB SUSCEPTIBLE 1
PCS: Protease Cleavage Site
PepMoV: Pepper Mottle Virus
PPV: Plum Pox Virus
PVY: Potato Virus Y
RNLs: RESISTANCE TO POWDERY MILDEW 8-like CC domain-containing NLRs
RPS5: RESISTANCE TO PSEUDOMONAS SYRINGAE 5
SMV: Soybean Mosaic Virus
TEV: Tobacco Etch Virus
TNLs: Toll/interleukin-1 receptor domain-containing NLRs
TuMV: Turnip Mosaic Virus
